# Giant scrotal elephantiasis of inflammatory etiology: a case report

**DOI:** 10.1186/1752-1947-1-23

**Published:** 2007-06-02

**Authors:** Stefan Denzinger, Elke Watzlawek, Maximilian Burger, Wolf F Wieland, Wolfgang Otto

**Affiliations:** 1 Department of Urology, University of Regensburg, Landshuterstraße 65, 93053 Regensburg, Germany

## Abstract

**Background:**

Scrotal lymphedema is rare outside endemic filariasis regions in Africa and Asia. It is of variable origin in the western world.

**Case presentation:**

We present a case of a 40-year-old European man with massive elephantiasis of the scrotum attributed to chronic inflammation of the lower urinary tract caused by urinary outlet obstruction and diabetes mellitus. The patient underwent subtotal scrotectomy saving penis, testes and spermatic cords and followed by scrotal reconstruction with adequate cosmetic and functional outcome.

**Conclusion:**

In this report we discuss a rare case of scrotal elephantiasis in an European patient, reflect on the etiology and the diagnostic and therapeutic approaches. Surgery can be successful even in giant scrotal elephantiasis.

## Background

Massive scrotal lymphedema, also termed elephantiasis, can be caused by obstruction, aplasia or hypoplasia of lymphatic vessels. It is usually caused by acquired infection e.g. lymphogranuloma venereum or filarial infestation with Wuchereria bancrofti. Scrotal elephantiasis is extremely rare outside endemic regions in Africa and India [1,2]. Occasionally it has been attributed to radiotherapy, neoplasm and lymphadenectomy [3,4].

Primary lymphedema i.e. congenital elephantiasis, is an extremely rare condition. Patients develop edema at adolescence without restriction to the external genitalia. In hereditary elephantiasis of the Meige type, lymphedema of the external genitalia occurs due to malformation of lymphatic vessels [4]. In some cases hidradenitis suppurativa and lichen sclerosus et atrophicus have also been described [5].

Scrotal elephantiasis is both emotionally distressing and physically disabling. Difficulties with hygiene, urinary incontinence, unesthetic appearance, loss of libido and immobility are severely debilitating symptoms. The etiology of the lymphedema usually determines the natural course and the therapeutic approach.

## Case presentation

### Case report

In April 2005, a 40-year-old Caucasian male was admitted to the Department of Urology of the University of Regensburg, Germany, with massive scrotal elephantiasis. He had been undergoing antibiotic treatment in another hospital for the previous three weeks.

The patient had a history of spina bifida with paralysis of the lower extremities, neurogenic bladder dysfunction, insulin dependent diabetes mellitus type 2 and slight mental retardation. Up to the age of 30 the patient was mobile by the use of a wheelchair, however the increasing size of his scrotum had made him bed-ridden for the past 10 years. Suprapubic cystostomy had been performed outside our institution ten years ago due to residual urine and recurrent urinary tract infections. There was no history of sexual contact, surgery, irradiation or travel.

**Figure 1:**
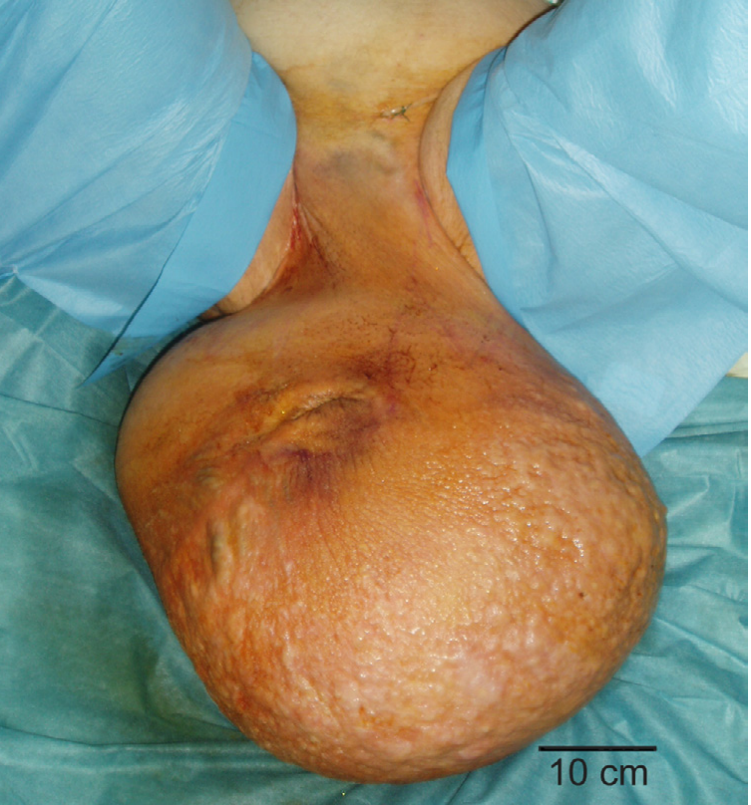
Preoperative view of the patient with massive scrotal elephantiasis.

On examination, the patient had a massively enlarged scrotum extending below his knees. The huge solid verrucous scrotal mass of 65 × 55 × 25 cm made it impossible to differentiate the anatomic structures (figure 1) and the urethral orifice emerged as a deep pit on the anterior surface of the mass. The scrotal skin was thickened and edematous hiding the penis. No inguinal adenopathy was found. Many superficial decubitus ulcers were found on the dorsal aspect of the scrotum. There was no accompanying swelling of the lower extremities. The testes and cords were not palpable, but no abnormalities were shown by ultrasonography. A CT scan of the abdomen indicated no cause of the condition. Laboratory testing, including human immunodeficiency virus, markers for testicular cancer, antibodies to schistosomes, Chlamydia trachomatis and filariae, were all in the normal range.

We decided to perform a subtotal scrotectomy with preservation of the penis and both testes and subsequent reconstruction of the scrotum by rotation flap. Primary closure of the wound was achieved.

**Figure 2:**
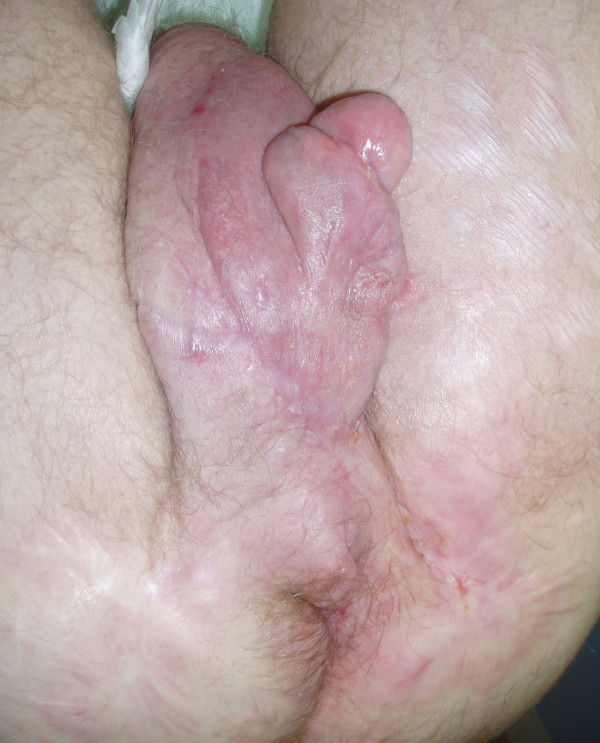
Appearance of the scrotum 12 weeks postoperatively.

The excised scrotal tissue weighed 11.6 kg. Grossly, the specimen contained multiple fluid-filled cysts. Histopathologic examination showed nonspecific chronic inflammation with areas of epidermal thickening and dermal fibrosis. Possibly due to the patient's diabetes mellitus, wound healing was impaired necessitating repeated wound debridement, vacuum assisted closure therapy for two weeks and the use of a mesh skin graft. Finally, wound healing was achieved with acceptable cosmetic results and only moderate distortion of the penis (figure 2).

### Discussion

Scrotal elephantiasis is a rare condition outside regions endemic for Chlamydia trachomatis or Wuchereria bancrofti. McDougal presented an overview of etiologic factors of lymphedema of the external genitalia in 2003. Besides infections, neoplasms or chronic inflammation, scrotal lymphedema is rarely caused by congenital conditions, e.g. the rare Meige syndrome leading to malformation of lymphatic vessels of the external genitalia [3].

In this case extensive diagnostics indicated no other cause than chronic lower urinary tract infection and thus the case presented here was attributed to inflammation. Residual urine related to the patient's neurogenic bladder led to chronic inflammation that was further enhanced by his type 2 diabetes mellitus. Postoperatively, even after meticulous histopathologic and electron microscopic preparation of the tissue, no other etiologic evidence was found apart from nonspecific inflammatory reactions.

Some cases of scrotal lymphedema warrant elimination of the cause of the disease e.g. by antibiotic therapy. Usually there is no permanent damage to the skin, lymphatic or subcutaneous tissue [1,6,7]. However, in a case of persistent scrotal lymphedema, irreversible damage of the involved tissue can occur with the danger of necrotising fasciitis.

In this case we finally opted for surgical intervention. In extensive disease, complete excision of all elephantoid tissue, preferably saving the penis, spermatic cord and testes, is appropriate [2,8,9]. In accordance with the desires of the patient we preserved the spermatic cords and both testes despite the extent of the disease. If available, scrotal flaps are most suitable for reconstruction of the scrotum. Medial thigh flaps can be used in the absence of adjacent scrotal tissue. Mesh skin graft is widely accepted for use in penile skin defects [10].

Despite protracted wound healing taking 10 weeks until final closure, the patient gained considerably in quality of life. The patient now is able to move himself by wheelchair again. After a follow-up period of 18 months the patient's erectile function was reestablished enabling sexual intercourse. This case shows that surgical therapy can provide good functional and cosmetic results even in massive scrotal elephantiasis.

## Conclusion

Scrotal lymphedema is a rare syndrome outside regions of endemic Chlamydia trachomatis or Wuchereria boncrofti like Africa or Asia. Scrotal lymphedema in the western world is of variable origin. We present one European patient with scrotal lymphedema caused by chronic lower urinary tract infection and discuss the diagnostical and therapeutical approach. In the case presented here extensive excision of elephantoid tissue saving penis, spermatic cord and testes was performed with adequate cosmetic and functional results.

## Competing interests

The author(s) declare that they have no competing interests.

## Authors' contributions

SD and WO drafted the manuscript, EW and MB helped to draft the manuscript. WFW supervised treatment and draft of the manuscript. All authors have read and approved the final manuscript.

## References

[B1] Nelson RA Alberts GL King LE Jr Penile and scrotal elephantiasis caused by indolent Chlamydia trachomatis infection Urology 2003 61 224 10.1016/S0090-4295(02)02078-2 12559311

[B2] Kuepper D Giant scrotal elephantiasis Urology 2005 65 389 10.1016/j.urology.2004.08.051 15708070

[B3] Mc Dougal WS Lymphedema of the external genitalia J Urol 2003 170 711 716 10.1097/01.ju.0000067625.45000.9e 12913680

[B4] Tammer ME Plogmeier K Schneider W Surgical therapy of scrotal edema in elephantiasis congenita hereditaria (Meige type) Urol A 2002 41 493 495 10.1007/s00120-001-0156-0 12426869

[B5] Wille S Niesel T Breul J Hartung R Elephantiasis of the legs with lichen sclerosus et atrophicus of the penis and scrotum J Urol 1997 157 2262 10.1016/S0022-5347(01)64747-5 9146642

[B6] Bernhard P Magnussen P Lemnge MM A randomized, double-blind, placebo-controlled study with diethylcarbamazine for the treatment of hydrocele in an area of Tanzania endemic for lymphatic filariasis Trans R Soc Trop Med Hyg 2001 95 534 536 10.1016/S0035-9203(01)90031-8 11706668

[B7] Makunde WH Kamugisha LM Massaga JJ Makunde RW Savael ZX Akida J Salum FM Taylor MJ Treatment of coinfection with bancroftian filariasis and onchocerciasis: a safety and efficacy study of albendazole with ivermectin compared to treatment of single infection with bancroftian filariasis Filaria J 2003 2 15 10.1186/1475-2883-2-15 14613509 PMC293471

[B8] Apesos J Anigian G Reconstruction of penile and scrotal lymphedema Ann Plast Surg 1991 27 570 573 10.1097/00000637-199112000-00010 1793244

[B9] Slama A Jaidane M Elleuch A Ben Sorba N Yacoubi MT Mosbah AF Surgical treatment of penile-scrotal elephantiasis Prog Urol 2002 12 337 339 12108357

[B10] Costa-Ferreira A Martins A Amarante J Silva A Reis J Giant penoscrotal elephantiasis Eur J Plast Surg 1999 22 397 399 10.1007/s002380050222

